# A maternal low-protein diet during gestation induces hepatic autophagy-related gene expression in a sex-specific manner in Sprague-Dawley rats

**DOI:** 10.1017/S0007114521003639

**Published:** 2022-08-28

**Authors:** Mingzhu Cai, Jie Zhang, Hong Chen, Yuan-Xiang Pan

**Affiliations:** 1 Department of Food Science and Human Nutrition, University of Illinois at Urbana-Champaign, Urbana, IL 61801, USA; 2 Division of Nutritional Sciences, University of Illinois at Urbana-Champaign, Urbana, IL, USA; 3 Department of Medicine, Imperial College Hammersmith Campus, London, UK

**Keywords:** Maternal protein deficiency, Genes, Autophagy, Transcription factor, Sex difference

## Abstract

This study investigates the mechanism by which maternal protein restriction induces hepatic autophagy-related gene expression in the offspring of rats. Pregnant Sprague-Dawley rats were fed either a control diet (C, 18 % energy from protein) or a low-protein diet (LP, 8·5 % energy from protein) during gestation, followed by the control diet during lactation and post-weaning. Liver tissue was collected from the offspring at postnatal day 38 and divided into four groups according to sex and maternal diet (F-C, F-LP, M-C and M-LP) for further analysis. Autophagy-related mRNA and protein levels were determined by real-time PCR and Western blotting, respectively. In addition, chromatin immunoprecipitation (ChIP) was performed to investigate the interactions between transcription factors and autophagy-related genes. Protein levels of p- eukaryotic translation initiation factor 2a and activating transcription factor 4 (ATF4) were increased only in the female offspring born to dams fed the LP diet. Correlatively, the mRNA expression of hepatic autophagy-related genes including *Map1lc3b, P62/Sqstm1, Becn1, Atg3, Atg7 and Atg10* was significantly greater in the F-LP group than in the F-C group. Furthermore, ChIP results showed greater ATF4 and C/EBP homology protein (CHOP) binding at the regions of a set of autophagy-related genes in the F-LP group than in the F-C group. Our data demonstrated that a maternal LP diet transcriptionally programmed hepatic autophagy-related gene expression only in female rat offspring. This transcriptional programme involved the activation of the eIF2*α*/ATF4 pathway and intricate regulation by transcription factors ATF4 and CHOP.

Maternal dietary protein restriction is known to affect fetal development^(1,2)^ profoundly. Observational and experimental studies have shown that fetal growth is most vulnerable to maternal diets during the first trimester of gestation^(1,2,3,4)^. The gestational protein restriction rat model, with 5–10 % protein compared with the 20 % protein in the control diet, has been one of the most widely researched models^(5)^. Gestational low-protein (LP) diets can impair fetal development by programming metabolic response pathways and regulating the expression of stress-response genes in offspring and may lead to long-term physiological consequences. For instance, a gestational LP diet could alter the expression of a set of hepatic genes and induced an altered metabolic phenotype in the liver of offspring^(6,7)^, which may provide a mechanism for the impaired lipid and carbohydrate metabolism induced by maternal protein restriction observed in later life^(8,9)^. Investigating these metabolic pathways and stress-induced genes in the offspring’s response to the maternal LP environment could improve the understanding of their lifelong phenotypic changes.

Dietary protein restriction is associated with numerous metabolic responses, including the activation of the eukaryotic translation initiation factor 2 (eIF2a)/activating transcription factor 4 (ATF4) pathway^(10)^. This pathway has been well characterised in cell culture models by amino acid limitation^(11,12,13)^. The initial steps are the accumulation of tRNA and the phosphorylation of serine 51 of the *α* subunit of eIF2a by GCN2 kinase. Then, the increased phosphorylation of eIF2*α* inhibits general protein synthesis but activates the translation of ATF4. ATF4, a master regulator of stress-induced genes, plays a key role in adaptation to nutrient stress by regulating the transcription of these genes. For instance, the expression of *Atf3* and *Chop* can be induced by ATF4, thus upregulating the amino acid response (AAR) pathway, which in turn affects the protein metabolism that helps to adapt to the LP environment^(14)^. Recent studies have shown that the AAR pathway can also be triggered in response to a maternal LP diet in dams, placenta and pups^(15,16)^. Strakovsky *et al.* showed that a gestational LP diet could activate the eIF2a/ATF4 pathway and further increase the expression of AAR downstream genes such as *Atf3* and *Chop* in the placenta^(15)^. Moreover, the activation of the AAR pathway has been found to be induced in the skeletal muscles of both dams and pups by a gestational LP diet^(16)^. These studies suggest that the eIF2a/ATF4 pathway can play an essential role in adapting to maternal protein restriction by programming the AAR pathway. However, more metabolic pathways might also be involved and need to be discovered.

The eIF2a/ATF4 pathway regulates stress-induced responses during maternal protein restriction. Among stress responses, autophagy pathways have attracted extensive interest. Autophagy is a cellular process that degrades cytoplasmic proteins and organelles via lysosomal pathways upon nutrient stress^(17,18)^. By recycling amino acids and other nutrients from soluble proteins and partial organelles in the cytoplasm, autophagy is involved in protein metabolism and basic metabolic processes to maintain normal energy levels and intracellular homoeostasis^(17)^. Recent studies have found that activated eIF2a/ATF4 up-regulates autophagy-related gene expression. B’Chir *et al.* revealed that the activation of the eIF2a/ATF4 pathway is required for autophagy-related gene expression in amino acid-starved cells^(11)^. Amino acid deprivation induces eIF2*α* phosphorylation and activates the expression of ATF4 and C/EBP homology protein (CHOP), which increases the transcription of a set of autophagy-related genes. These activated genes may contribute to the formation, maturation and function of autophagosomes^(11)^. *Wang et al.* were the first to report that a gestational LP diet induces autophagy-related gene expression in the skeletal muscles of rat offspring^(16)^. However, the role of eIF2a/ATF4 pathway in the increased expression of autophagy-related genes in the offspring and the specific target genes of ATF4 and CHOP are not fully understood.

The present study investigated hepatic autophagy-related gene expression regulated by the eIF2*α*/ATF4 pathway in both male and female offspring of dams fed a gestational LP diet. The hypothesis states that maternal protein restriction during gestation will induce metabolic stress responses in the liver of offspring, which occurs with a transcriptional programme of autophagy-related gene expression in a sex-specific manner to adapt to the nutrient-deprivation stress.

## Methods

### Animal treatments

**Fig. 1.**
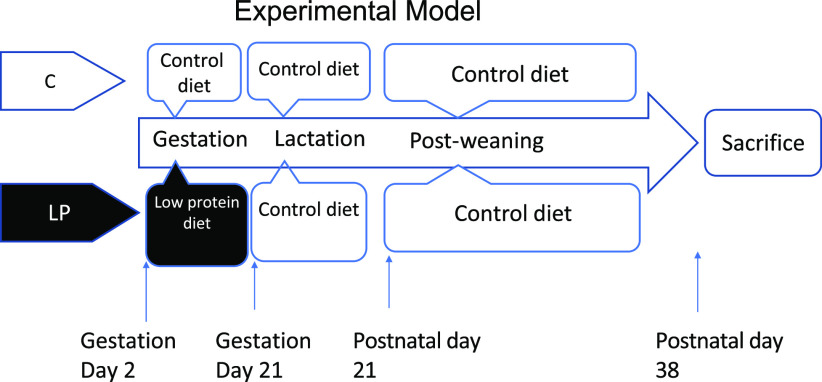
The experimental design of animal study. C, pregnant rat dams fed on a control diet; LP, pregnant rat dams fed on a low-protein diet.

Timed-pregnant Sprague-Dawley rats were purchased from Charles River Laboratories and were individually housed in standard polycarbonate cages in a humidity- and temperature-controlled room with free access to food and water on a 12-h light/12-h dark cycle. They were fed either a control diet (C group, 18 % energy from protein) or a LP diet (LP group, 9 % energy from protein) (Bioserve) from gestation day 2 during gestation. The experimental diets were designed according to well-established diet formula^(6,14,19)^ as listed in Table 1. All dams were fed with the same control diet after delivery during lactation. After weaning, all pups were kept on the same control diet until sacrifice at postnatal day 38 (Fig. 1). One pup per dam was used for further experiments; therefore, our experimental unit was a single animal (*n* 8 for each group). All pups were individually housed in standard polycarbonate cages in a humidity- and temperature-controlled room with free access to food and water on a 12-h light/12-h dark cycle. The pups were killed by CO_2_ asphyxiation at postnatal day 38 following 12 h of fasting. The liver from each pup was collected, snap-frozen in liquid N_2_ and stored at –80°C until it can be processed. The animal protocol for this study was approved by the International Animal Care and Use Committee at the University of Illinois.

**Table 1.** Nutrient composition of the experimental diets

Ingredients	C, g/kg	LP, g/kg
Maize starch	405	465
Sucrose	213	243
Casein	180	90
Maize oil	100	100
Cellulose	50	50
Mineral mix*	35	35
Vitamin mix†	10	10
d, l-methionine	5	5
Choline	2	2
Folic acid	0·001	0·001
Macronutrient composition	C, % of total energy	LP, % of total energy
Carbohydrate	66 %	75 %
Protein	18 %	9 %
Fat	16 %	16 %
Total energy, kcal/kg	3868	3868

* Mineral mix: calcium phosphate dibasic 11·3 g/kg; sodium chloride 1·7 g/kg; potassium citrate monohydrate 5·0 g/kg; potassium sulphate 1·2 g/kg; magnesium sulphate 0·5 g/kg; magnesium carbonate 0·1 g/kg; ferric citrate 0·1 g/kg; zinc carbonate 36·2 mg/kg; cupric carbonate 6·8 mg/kg; potassium iodate 0·2 mg/kg; sodium selenite 0·2 mg/kg; chromium potassium sulphate 12·5 mg/kg.

† Vitamin mix: thiamine hydrochloride 2·4 mg/kg; riboflavin 2·4 mg/kg; pyridoxine hydrochloride 2·8 mg/kg; nicotinic acid 12·0 mg/kg; d-calcium pantothenate 6·4 mg/kg; biotin 0·01 mg/kg; cyanocobalbumin 0·003 mg/kg; retinyl palmitate 6·4 mg/kg; dl-μ-tocopherol acetate 79·9 mg/kg; cholecalciferol 1·0 g/kg; menaquinone 0·02 mg/kg.

### RNA isolation and real-time quantitative PCR

Liver tissue samples were ground in liquid N_2_ in a mortar and pestle, and total RNA was isolated using TRIZOL reagent (Sigma-Aldrich) as described previously^(16)^. Total mRNA was used for DNA synthesis using the High Capacity cDNA Reverse Transcription Kit (Applied Biosystems), which was performed in a thermocycler (Applied Biosystems) at 25°C for 10 min, 37°C for 2 h and 85°C for 5 s. PCR was performed with a 96-well plate using a SYBR Green PCR Master mix (Applied Biosystems) on the StepOnePlus Real-Time PCR System (Applied Biosystems). The reaction conditions were 95°C for 15 min, 95°C for 15 s and 60°C for 60 s for forty cycles. Table 2 lists the primer sequences and source for each primer used in this study. After quantification, the ribosomal protein L7a was used to normalise all the mRNA data.

**Table 2.** List of primers

Gene (ensemble no. transcript ID)	Sequence		
*L7a* (ENSRNOT00000006754)	mRNA	Forward +64F	GAG GCC AAA AAG GTG GTC AAT CC
		Reverse + 127R	CCT GCC CAA TGC CGA AGT TCT
*GAPDH* (ENSRNOG00000018630)	mRNA	Forward +220F	CTC TAC CCA CGG CAA GTT CAA CG
		Reverse + 311R	CTC GCT CCT GGA AGA TGG TGA TG
*Actb* (ENSRNOG00000034254)	mRNA	Forward +451F	CTC TAC CCA CGG CAA GTT CAA CG
		Reverse + 526R	CTC GCT CCT GGA AGA TGG TGA TG
*Atf3* (ENSRNOT00000005085)	mRNA	Forward +688F	AGA AGG AAC ATT GCA GAG CTA AGC
		Reverse + 767R	TGG GGT GGA AAA GGA GGA TTC
*Chop* (ENSRNOG00000006789)	mRNA	Forward +636F	CTC TGA TCG ACC GCA TGG TCA G
		Reverse + 731R	TGG CGT GAT GGT GCT GGG
*Map1lc3b* (ENSRNOT00000051352)	Genomic DNA	Forward −244F	AGC CAG GAG CAG AAG ATG AA
		Reverse − 156R	TGA GTC GGC TTC CCA AAA
	mRNA	Forward +626F	GTG TTG TGG AAG AAT GCC
		Reverse + 726R	TCA CCC TTG TAT CGC TCT
*Atg3* (ENSRNOT00000002906)	Genomic DNA	Forward −125F	GGC CAG TTC TCC CTG GAA GCT AT
		Reverse − 56R	CCC GCT TAA CTC CTG GTT ACC AAT
	mRNA	Forward +432F	AGT CCA CCA CTG TCC AAC AT
		Reverse + 505R	TCC CTG TTG GTA GAT ATG CC
*Becn1* (ENSRNOT00000023477)	Genomic DNA	Forward −1138F	GTG TGA GCA TAT CTG TTC CTG T
		reverse − 1069R	AGG TAA GCG AAA TGT GGT TC
	mRNA	Forward +1031F	TGC GAC CTT CCA TAT CTG
		Reverse + 1095R	AGC GAC CCA GTC TGA AAT
*P62/Sqstm1*(ENSRNOT00000059255)	Genomic DNA	Forward −175F	TGG TGA GAC CTG AGT TGC TG
		Reverse − 89R	CTG CTC ATC GGG GAA TGA
	mRNA	Forward +524F	ATC TTT CCC AAC CCC TTT G
		Reverse + 597R	CCA TGT TTC AGC TTC CGA A
*Atg7* (ENSRNOT00000067532)	Genomic DNA	Forward −973F	CCC CAG CTG ACT CTG GGA GAA
		Reverse − 884R	GGG TTG GGC ACA CAC TCA GGT
	mRNA	Forward +379F	CCC ATG CTC CTC AAC AAG TTT C
		Reverse + 458R	GGG CAG CAA AAC CAG TAG TAG AAG
*Atg5* (ENSRNOT00000057078)	Genomic DNA	Forward −911F	TTA CTG CCC TTT CCA CTC TGT C
		Reverse − 840R	TTT GCT AAC TGA GGT CAG GGA C
	mRNA	Forward +546F	AGAAGTCTGTCCTTCCGCAGTCG
		Reverse + 612R	CCC GTG AAT CAT CAC CTG GCT
*Atg10* (ENSRNOT00000021941)	Genomic DNA	Forward −543F	CCT GTT TAA AGG ACC AGA CT
		Reverse − 469R	AGG AGG AAA AGA GTC ATA GG
	mRNA	Forward +100F	CCA GAC ATT CAC AAC AGA TAG GC
		Reverse + 164R	AGC CAT CAG AAC ATT CCT TG

### Protein isolation and Western blotting

Protein concentrations were determined by the Lowry assay. Western blotting was performed as described by Wang *et al.*^(16)^. Frozen liver tissue (50 mg) from offspring was ground and suspended in 400 μl of protein sample buffer with a 1X proteinase inhibitor (Roche Applied Science) and phosphatase inhibitor cocktails 1 and 2 (Sigma-Aldrich). Each diluted sample (30 μg) was size-fractionated by 12 % SDS-PAGE, transferred to a 0·2-μm PVDF membrane (Bio-Rad) and blocked in 10 % non-fat dry milk in Tris Buffered Saline with Tween for 1 h at room temperature. Antibodies against target proteins were incubated with the membrane in 10 % non-fat dry milk at a 1:1000 dilution at room temperature for 1 h. Blots were incubated in Super Signal West Dura Extended Duration Substrate (Pierce) for 5 min, and then the signals were detected and analysed by Bio-Rad ChemiDoc and Quantity One software. The antibodies are listed in Table 3.

**Table 3.** List of antibodies

Name	Modification	Cat. number	Lot no.	Company name
Actin (I-19)-R		sc-1616-R	C0907	Santa Cruz Biotechnology, Inc, Santa Cruz, CA
CREB-2 (C-20)		sc-200	H2008	Santa Cruz Biotechnology, Inc, Santa Cruz, CA
ATF4 (D4B8)		11815S	Lot 4	Cell Signaling Technology, Inc, Danvers, MA
Normal rabbit IgG		sc-2027	F1608	Santa Cruz Biotechnology, Inc, Santa Cruz, CA
p-eIF2*α*	p-S51	9721L	Lot 15	Cell Signaling Technology, Inc, Danvers, MA
Total eIF2*α*		9722	Lot 11	Cell Signaling Technology, Inc, Danvers, MA
LC3A/B		4108S	Lot 3	Cell Signaling Technology, Inc, Danvers, MA
Beclin 1		3495S	Lot 2	Cell Signaling Technology, Inc, Danvers, MA
CHOP		2895S	Lot 11	Cell Signaling Technology, Inc, Danvers, MA

### Chromatin immunoprecipitation

To investigate the roles of the transcription factors ATF4 and CHOP in regulating autophagy-related gene expression, chromatin immunoprecipitation analysis was performed according to a modified protocol^(20)^ with magnetic beads as published previously^(21)^ for this study. Briefly, 200-mg frozen liver samples from offspring were ground in liquid N_2_ and suspended in 50 ml of PBS. Protein-DNA cross-linking was performed by 37 % formaldehyde in 50 ml of PBS buffer for 10 min at room temperature. The cross-linking reaction was stopped with 2 m glycine. The following steps are the same as described by Strakovsky *et al.*^(21)^. Purified DNA was quantified by real-time PCR (the primers are listed in Table 2).

### Statistical analysis

Results are presented as the mean values with their standard error of mean. Food intake and body weight were analysed by repeated-measures ANOVA. mRNA levels, protein levels and chromatin immunoprecipitation data were analysed using two-way ANOVA to look at the effects of maternal diets, sex and their interactions on the hepatic responses in the offspring, specially changes in the mechanisms that regulate autophagy-related gene expression (F-C and F-LP, M-C and M-LP, sample size *n* 8 for each). When a significant interaction was observed, a Bonferroni post hoc test was conducted to determine the effect of maternal diet on female (F-C *v*. F-LP) and male (M-C *v*. M-LP) offspring separately. All analyses were conducted in Prism 7. Differences were considered significant at *P* < 0·05 for all comparisons.

The sample size was calculated based on AAR gene expression data from a previous study^(16)^ to achieve 80 % statistical power and 0·05 two-sided significance level.

## Result

### Physiological changes and growth potentials

**Fig. 2.**
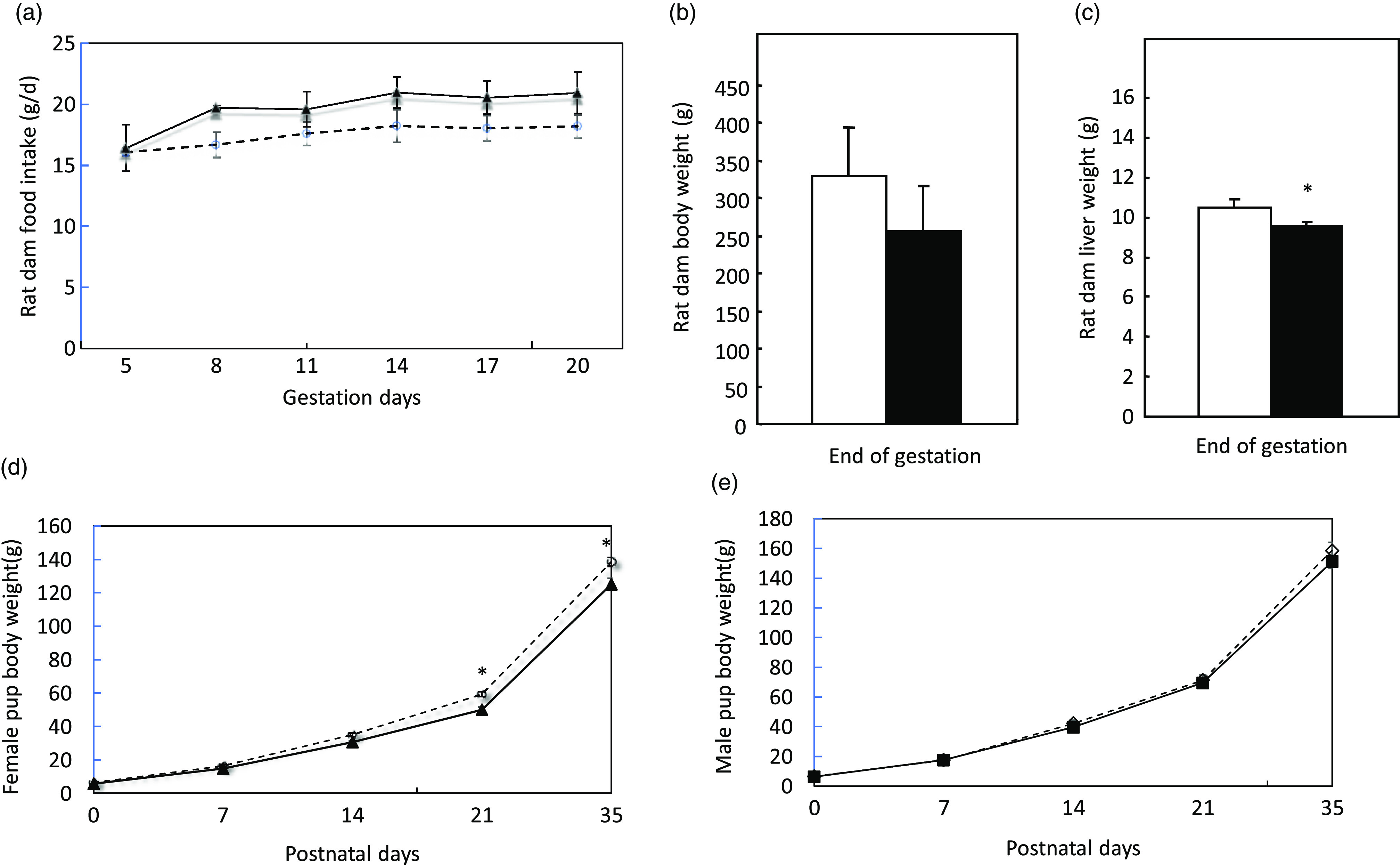
Food intake (a), body weight (b), liver weight (c) of pregnant dams and body weight of female (d) and male pups (e) in the study. Values are reported as the mean values with their standard error of mean, *n* 8. *P < 0·05 analysed by repeated-measures ANOVA. C, pregnant dams fed on a control diet; LP, pregnant dams fed on a low-protein diet. (a) 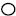, C; 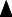, LP. (b) 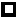, C; 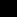, LP. (c) 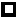, C; 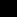, LP. (d) 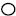, C; 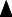, LP. (e) 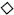, C; 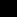, LP.

Pregnant dams were obtained on day 2 of gestation. Fig. 2(a) shows dams’ daily food intake from gestation day 3 to day 21, at which they gave birth. There were no differences in the daily food intake and body weight (Fig. 2(b)) at the end of gestation between dams in the control and low protein groups. However, dams’ liver weights in the low protein group were slightly but significantly lower than those in the control group (Fig. 2(c)). At birth, pups in the control group weighed similar to pups in the LP group. As the pups grew to 21 d, the average body weight of female pups in the LP group was significantly lower than those in the control group (Fig. 2(d)). The average weight of male offspring in the LP group was not significantly different from the control pups (Fig. 2(e)). Liver weight and food intake did not differ between the control and LP pups (not shown).

### Expression of amino acid response pathway-related genes

**Fig. 3.**
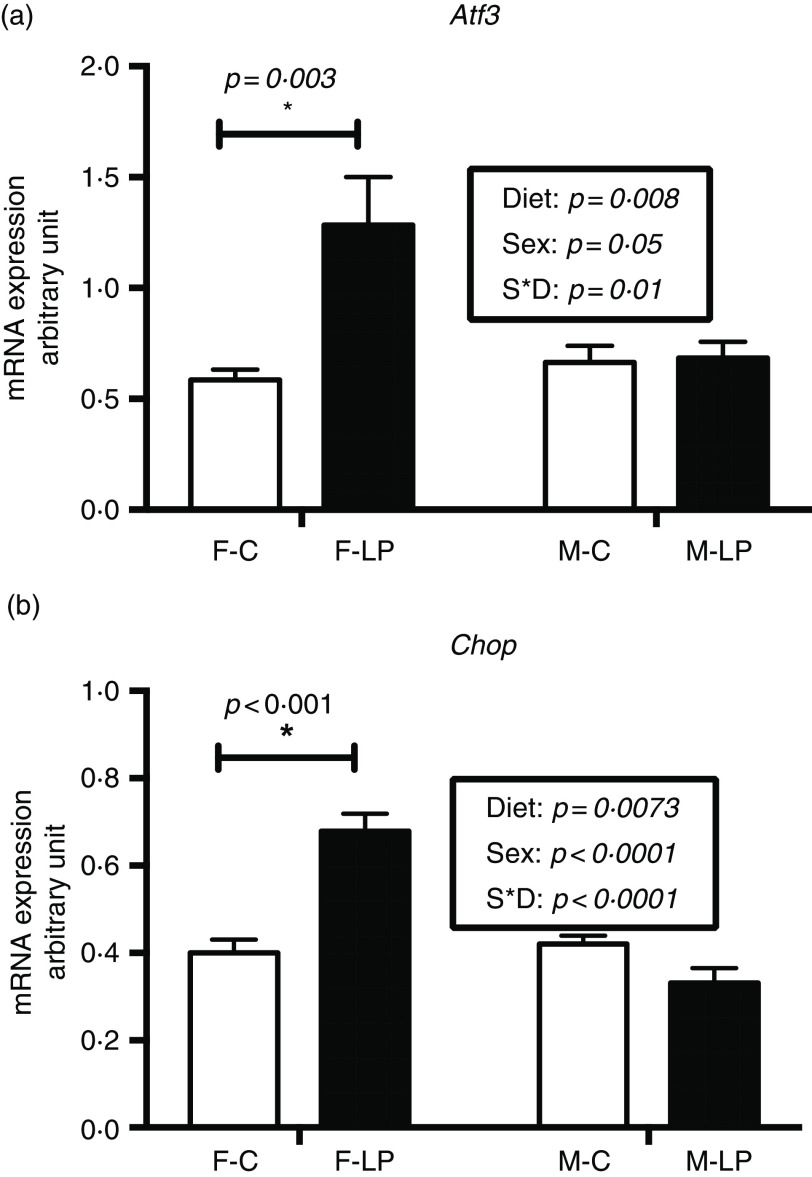
A maternal LP diet induces the mRNA expression of *Atf3* (a) and *Chop* (b) only in the liver of female offspring. All data were normalised to the expression level of the housekeeping genes *L7a*. Values are reported as the mean values with their standard error of mean, *n* 8. *P < 0·05 by post hoc Bonferroni test. *Atf3*, activating transcription factor 3; *Chop*, gene encoding C/EBP homology protein; *L7a*, gene encoding 60S ribosomal protein. F-C, female offspring born to the dams of control diet; F-LP, female offspring born to the dams of low-protein diet; M-C, male offspring born to the dams of control diet; M-LP, male offspring born to the dams of low-protein diet. (a) 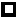, C; 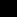, LP. (b) 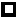, C; 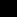, LP.

Two-way ANOVA demonstrated a significant interaction between maternal diet and offspring sex. Post hoc analysis showed that two markers of the AAR pathway, Atf3 (*P* = 0·003) and Chop (*P* < 0·001), exhibited significantly greater expression in the F-LP pups than in the F-C pups. However, there was no significant difference between the M-C and the M-LP pups (Fig. 3).

### Activation of the eukaryotic translation initiation factor 2/activating transcription factor 4 pathway

**Fig. 4.**
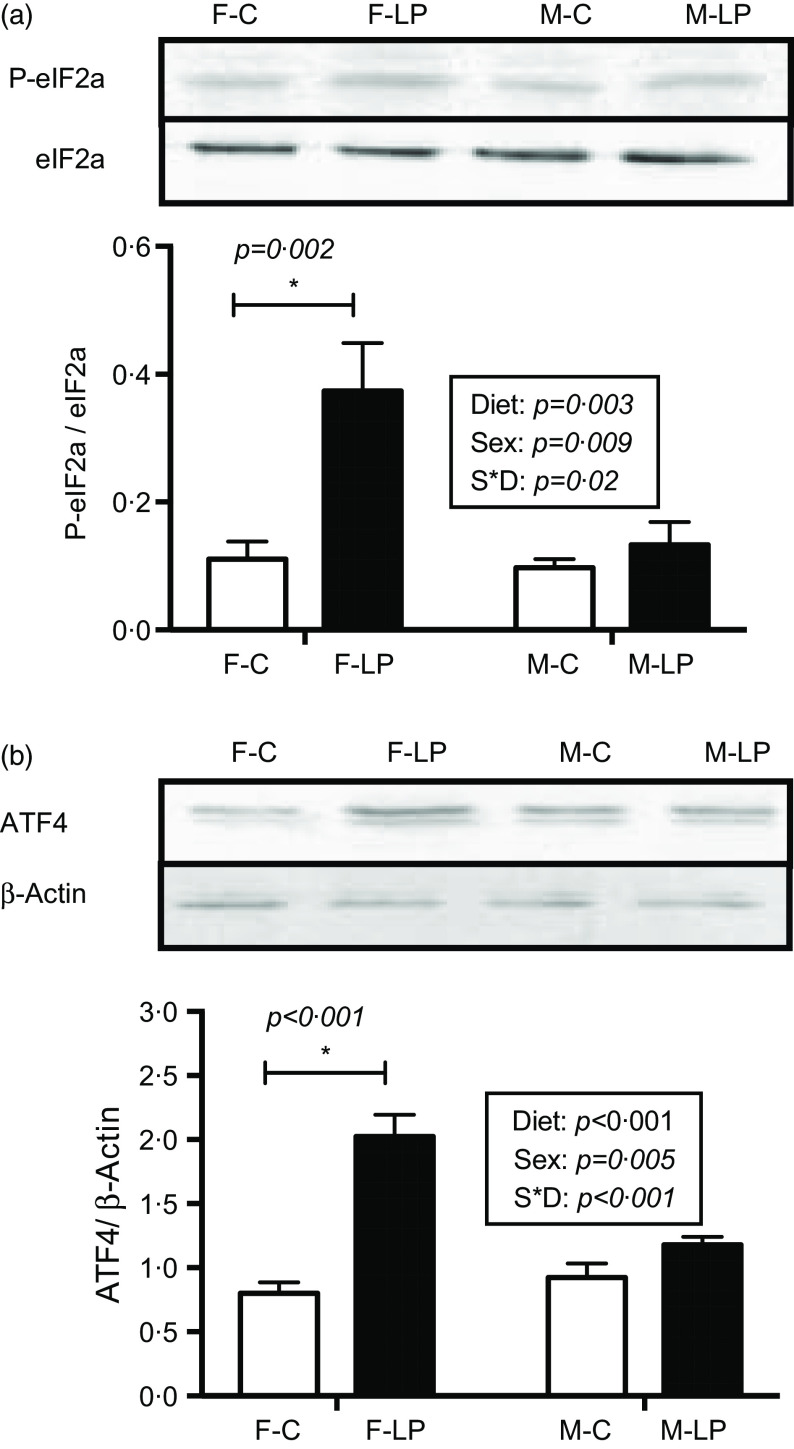
The maternal LP diet activates the protein levels of p-eIF2a (a) and ATF4 (b) in the female offspring. Total eIF2a was used to normalise the phosphorylation of eIF2a, and actin was used to normalise the protein levels of ATF4. Values are reported as the mean values with their standard error of mean, *n* 8. *P < 0·05 by post hoc Bonferroni test. p-eIF2a, phosphorylated eukaryotic translation initiation factor 2a; ATF4, activating transcription factor 4. F-C, female offspring born to the dams of control diet; F-LP, female offspring born to the dams of low-protein diet; M-C, male offspring born to the dams of control diet; M-LP, male offspring born to the dams of low-protein diet. (a) 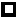, C; 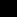, LP. (b) 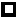, C; 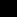, LP.

The eIF2a/ATF4 pathway was activated in the livers from the F-LP pups (Fig. 4). Two-way ANOVA showed a significant interaction between maternal diet and offspring sex. Therefore, a post hoc analysis was conducted and indicated that the protein levels of p-eIF2*α* (*P* = 0·002) and ATF4 (*P* < 0·001), which regulate the transcription of autophagy-related genes, were greater in the livers from the F-LP offspring than in those from the F-C offspring. The pathway was not activated in the livers from M-C and M-LP offspring (Fig. 4).

### Expression of autophagy-related gene expression

**Fig. 5.**
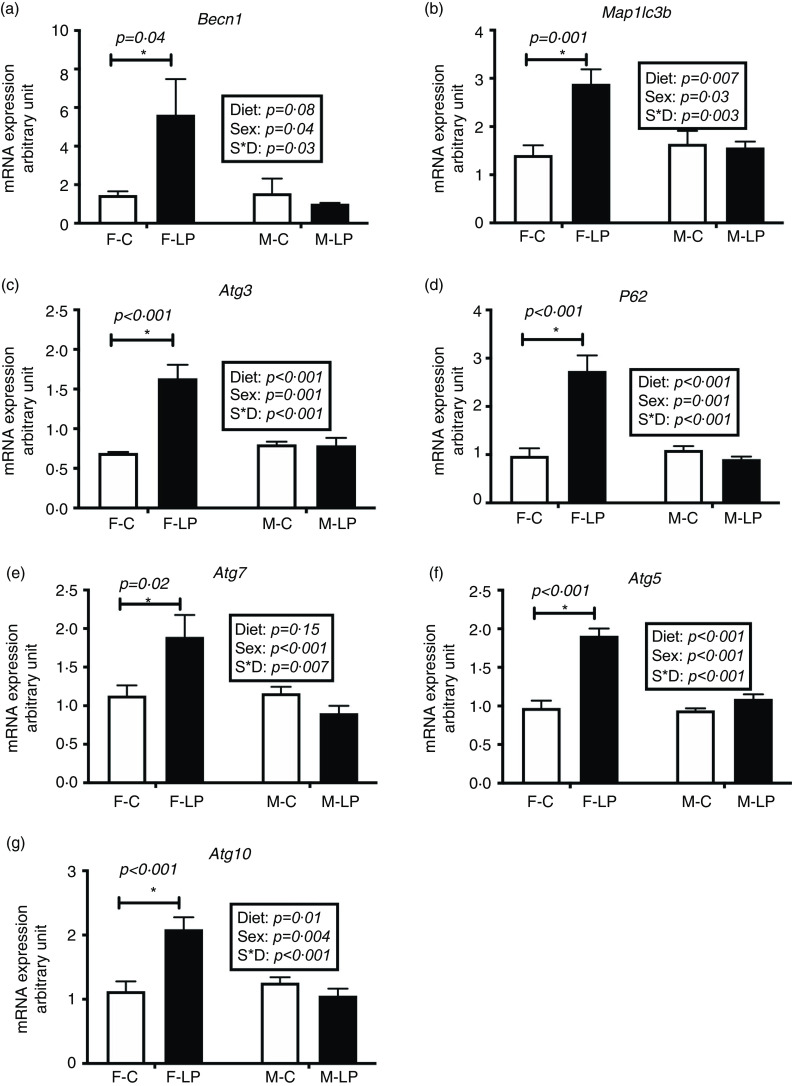
The mRNA expression of hepatic autophagy-related genes including *Becn1* (a), *Map1lc3b* (b), *Atg3* (c), *P62* (d), *Atg7* (e), *Atg5* (f) and *Atg10* (g) was greater significantly in the female rat offspring of dams fed a maternal LP diet than those of dams fed a control diet. All data were normalised to the mRNA expression level of *L7a*. Values are reported as the mean values with their standard error of mean, *n* 8. *P < 0·05 by post hoc Bonferroni test. *L7a*, gene encoding 60S ribosomal protein. F-C, female offspring born to the dams of control diet; F-LP, female offspring born to the dams of low-protein diet; M-C, male offspring born to the dams of control diet; M-LP, male offspring born to the dams of low-protein diet. (a) 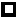, C; 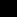, LP. (b) 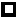, C; 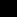, LP. (c) 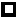, C; 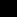, LP. (d) 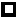, C; 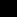, LP. (e) 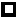, C; 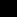, LP. (f) 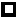, C; 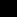, LP. (g) 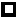, C; 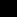, LP.

The female pups born to LP dams had increased mRNA levels of hepatic autophagy-related genes than those born to C dams (Fig. 5). There was a substantial interaction effect between maternal diet and offspring sex on the mRNA expression levels of a set of autophagy-related genes. In female offspring, the mRNA expression of *Becn1* (*P* = 0·04), *Atg3* (*P* < 0·001), *Map1lc3b* (*P* = 0·001), *P62/Sqstm1* (*P* < 0·001), *Atg7* (*P* = 0·02), *Atg5* (*P* < 0·001) and *Atg10* (*P* < 0·001) was significantly higher in the F-LP group than in the F-C group. In male offspring, there was no effect of maternal diet observed (Fig. 5).

### The role of activating transcription factor 4-C/EBP homology protein in autophagy-related gene expression

**Fig. 6.**
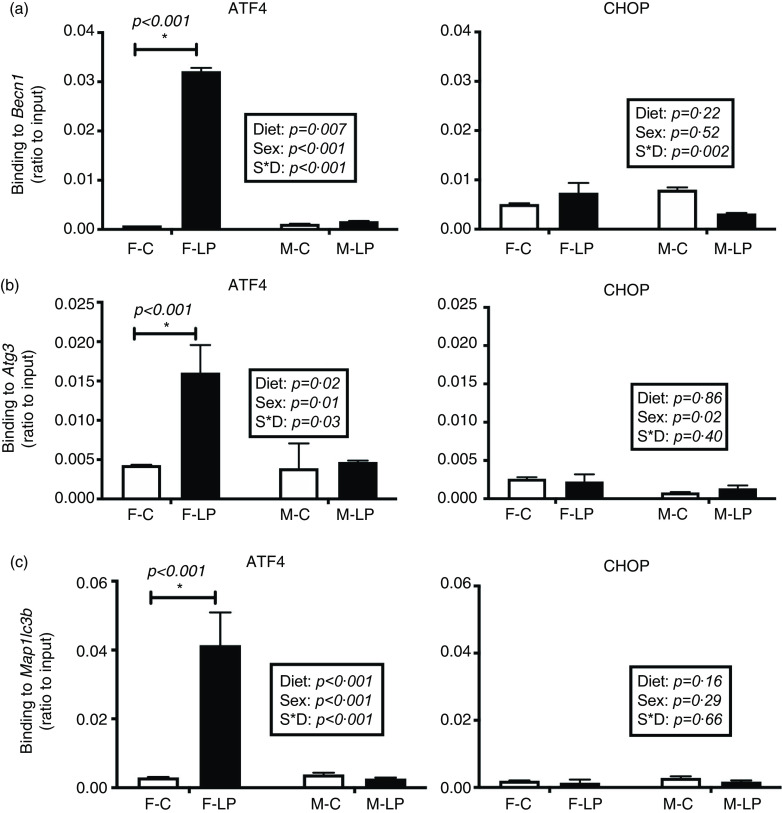
The transcription factor ATF4 but not CHOP bound to *Becn1* (a), *Atg3* (b) and *Map1lc3b* (c) in the liver of female offspring of dams fed a maternal LP diet. Values are reported as the mean values with their standard error of mean, *n* 8. *P < 0·05 by post hoc Bonferroni test. ATF4, activating transcription factor 4; CHOP, C/EBP homology protein. F-C, female offspring born to the dams of control diet; F-LP, female offspring born to the dams of low-protein diet; M-C, male offspring born to the dams of control diet; M-LP, male offspring born to the dams of low-protein diet. (a) 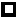, C; 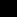, LP. (b) 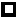, C; 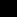, LP. (c) 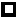, C; 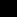, LP.

**Fig. 7.**
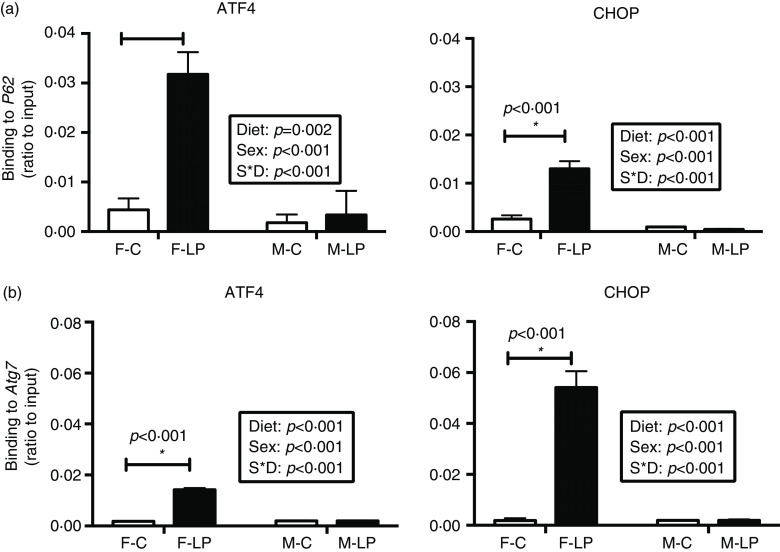
The binding of both ATF4 and CHOP to the autophagy-related genes *P62* (a) and *Atg7* (b) was induced in the liver of female offspring of dams fed a maternal LP diet. Values are reported as the mean values with their standard error of mean, *n* 8. *P < 0·05 by post hoc Bonferroni test. ATF4, activating transcription factor 4; CHOP, C/EBP homology protein. F-C, female offspring born to the dams of control diet; F-LP, female offspring born to the dams of low-protein diet; M-C, male offspring born to the dams of control diet; M-LP, male offspring born to the dams of low-protein diet. (a) 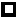, C; 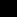, LP. (b) 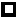, C; 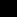, LP.

**Fig. 8.**
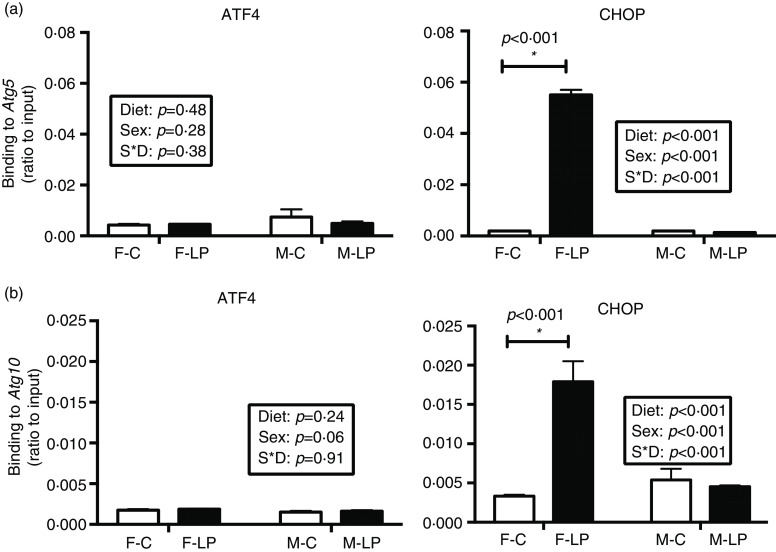
The maternal LP diet induced the transcription factor CHOP but not ATF4 to bind to *Atg5* (a) and *Atg10* (b) in the liver of female offspring. Values are reported as the mean values with their standard error of mean, *n* 8. *P < 0·05 by post hoc Bonferroni test. ATF4, activating transcription factor 4; CHOP, C/EBP homology protein. F-C, female offspring born to the dams of control diet; F-LP, female offspring born to the dams of low-protein diet; M-C, male offspring born to the dams of control diet; M-LP, male offspring born to the dams of low-protein diet. (a) 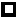, C; 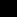, LP. (b) 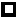, C; 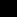, LP.

A post hoc test was conducted in female and male offspring separately due to a significant interaction between maternal diet and offspring sex. ATF4 and CHOP cooperatively regulated the transcriptional induction of autophagy-related genes in the livers in the female offspring. From the chromatin immunoprecipitation results, we found that: (1) *ATF4* but not CHOP bound at the regions of *Becn1* (*P* < 0·001), *Atg3* (*P* = 0·01) and *Map1lc3b* (*P* < 0·001) (Fig. 6); (2) both *ATF4* and CHOP bound to *P62/Sqstm1* (both *P* < 0·001) and *Atg7* (both *P* < 0·001) (Fig. 7); and (3) CHOP bound in the regions of *Atg5* (*P* < 0·001) and *Atg10* (*P* < 0·001) (Fig. 8). However, none of these bindings was observed differently in any male offspring (Figs. 6–8).

## Increased protein levels in the autophagy initiation

**Fig. 9.**
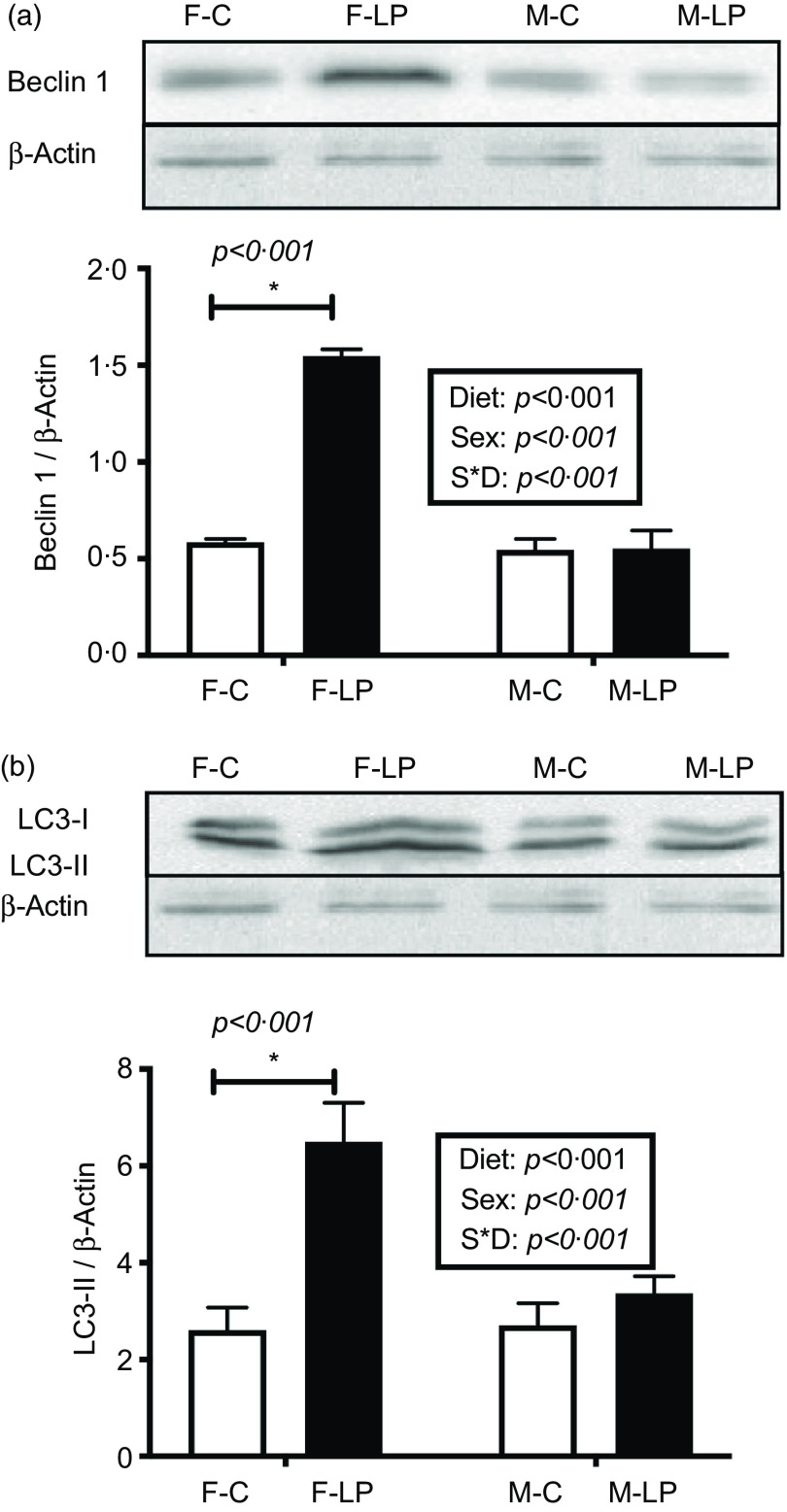
The protein levels of Beclin1 (a) and LC3-II (b) were induced by the maternal LP diet only in the liver of female offspring. All data were normalised to the protein levels of Actin. Values are reported as the mean values with their standard error of mean, *n* 5. *P < 0·05 by post hoc Bonferroni test. LC3, microtubule-associated protein 1 light chain 3. F-C, female offspring born to the dams of control diet; F-LP, female offspring born to the dams of low-protein diet; M-C, male offspring born to the dams of control diet; M-LP, male offspring born to the dams of a low-protein diet. (a) 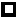, C; 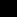, LP. (b) 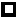, C; 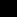, LP.

The protein levels of some of the genes in the autophagy pathway were detected in the female offspring exposed to a maternal LP diet (Fig. 9). A post hoc test was conducted within each sex due to a significant interaction between maternal diet and offspring sex. In female offspring, protein levels of Beclin 1 (*P* < 0·001) and LC3-II (*P* < 0·001), which are the primary indicators of the initiation in the autophagy pathway, were significantly higher in the F-LP group than in the F-C group. In male offspring, the maternal diet had no significant effect on these two protein levels (Fig. 9).

## Discussion

**Fig. 10.**
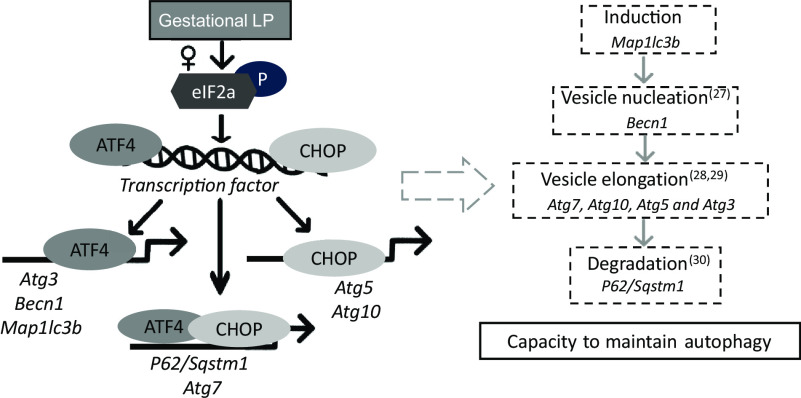
A schematic model of autophagy-related gene transcriptional programming by maternal protein restriction in the liver of female rat offspring. It was adapted and redrawn from B’Chir *et al.*^(11)^ with permission. In response to a low-protein diet during gestation, eIF2a was phosphorylated in the liver of female offspring, which further induced the transcription factors ATF4 and CHOP. ATF4 and CHOP then established a transcriptional programme of autophagy-related gene expression. The activated autophagy-related genes might result in an increased capacity to maintain autophagy by encoding autophagy-related proteins that can directly participate in the autophagic process. ATF4, activating transcription factor 4; CHOP, C/EBP homology protein.

Using an *in vivo* LP model, the present study demonstrates that maternal protein restriction during gestation affects the expression of autophagy-related genes in the liver of female offspring only. In response to a gestational LP diet, eIF2*α* was phosphorylated, which further activated ATF4. The activated eIF2*α*/ATF4 pathway induced autophagy-related gene expression through cooperative regulation of ATF4 and CHOP. A set of autophagy-related genes could be identified and divided into three classes according to their dependence on ATF4 and CHOP. These were only observed in the livers from female offspring but not from the male. This study provides the first direct evidence suggesting that maternal protein restriction during gestation induces hepatic autophagy-related gene expression in a sex-specific manner in the offspring. The induction of autophagy-related gene expression may result in an increased capacity to undergo autophagy in the liver of female offspring (Fig. 10).

Autophagy has been widely reported recently in different physiological conditions. However, understanding of the mechanisms that control autophagy pathways is limited, especially at the transcriptional level. This study focused on two major regulators, ATF4 and CHOP, which activate the transcription of the target genes *Atg5* and *Map1lc3b* during hypoxia^(22)^ and ER stress^(23,24)^. Nevertheless, there is limited research into their transcriptional roles in autophagy induced by nutrient stress. Furthermore, the downstream target autophagy-related genes have not been fully investigated. Recently, B’Chir *et al*. proposed a mechanism underlying autophagy-related gene expression regulated by eIF2*α*/ATF4 in mouse embryonic fibroblast cells upon amino acid starvation^(11)^. This study suggested that ATF4 and CHOP were required to induce the transcription of a set of autophagy-related genes. Three classes of autophagy-related genes were clustered according to their different dependences on ATF4 and CHOP^(11)^. Based on the autophagy transcriptional programme found by B’Chir, the present study further investigated the transcriptional roles of ATF4 and CHOP in a rat offspring model in which dams were fed on an LP diet during gestation. In the present study, cooperative regulation by ATF4 and CHOP resulted in three types of transcriptional programmes: (i) ATF4 but not CHOP bound to the promoter regions of *Map1lc3b, Atg3* and *Becn1* and up-regulated the transcription of these genes; (ii) both ATF4 and CHOP were observed to bind at the regions of *P62/Sqstm1* and *Atg7*, which further activated their gene expression; and (iii) CHOP binding at the corresponding promoter induced the gene expression of *Atg5* and *Atg10* without direct interaction with ATF4. This investigation of the ATF4/CHOP-regulated programme will improve the understanding of the transcriptional regulation of autophagy pathways.

Autophagy pathways involve induction, nucleation, formation, elongation and degradation of autophagosomes^(25,26,27,28,29,30)^. The increased protein levels of Beclin 1 and LC3-II, as well as the elevated mRNA expression of a set of autophagy-related genes in a sex-specific effect in our study, may suggest an increased capacity to undergo autophagy in the liver of female offspring (Fig. 10). However, the cause is not fully understood. This difference potentially originated from the placenta through sex-dependent epigenetic regulation^(31)^. Many diseases show alterations in autophagy and sex-associated differences^(32,33,34)^. Different degrees of autophagy depending on sex may be controlled by the redox-sensitive transcription factors, such as NF-κB and p53 in an organ-specific way^(34,35)^. Several hepatic genes could respond to maternal protein restriction in a sex-specific manner, including *G6PC*^(36)^, *PEPCK*^(37)^ and *11beta-HSD1*^(37)^. Hormonal regulation may also contribute to sex dimorphism, including oestrogen played an essential role in autophagy regulation in the liver and exhibited female-biased response^(38,39)^. Moreover, maternal protein restriction could impair androgen levels in male offspring^(40,41)^ and influenced liver metabolism^(42)^. The different features displayed in this study could be mainly due to hormonal regulators, as previously suggested^(43)^. However, how hormonal regulators influence ATF4 and CHOP binding to specific genes is largely unknown at this moment. It is also possible that timing for the postpartum analysis may be important. For example, males may show a similar response, but this response occurs earlier or later (before/after current postpartum analysis). More research must be conducted to investigate the role of epigenetics and sex hormones in offspring to understand how maternal nutrition differentially impacts males and females fully.

The sex-specific responses in hepatic autophagy-related genes may result in differential physiological outcomes and potential diseases. Autophagy-related genes have been linked to human cancers and other diseases. For instance, *Becn1* has been reported to be a tumour-suppressor gene. In a *Becn1*-mutant mouse model, the development of hepatitis B virus-induced premalignant lesions was significantly accelerated^(44)^. Another example is the role of *Atg7* in lipid metabolism in the liver of mice. In *Atg7*-knockout mice, a massive accumulation of lipid droplets has been found in the liver, suggesting that without *Atg7*, mice cannot undergo autophagy to degrade unnecessary lipids and have an elevated risk of non-alcoholic fatty liver disease^(45)^. Several sex-biased diseases can be explained to some extent by sex-specific autophagy-related gene expression. For example, differences in sex-specific autophagy-related gene expression have been found in cardiac samples from both mice and humans, potentially explaining the disparity in heart disease between the sexes found in clinical studies^(46)^. Our findings highlight the sex difference in autophagy-related gene expression in rat offspring of dams exposed to gestational protein restriction, suggesting that male and female offspring might have different hepatic conditions. Future studies should investigate the hepatic physiological outcomes in male and female offspring experiencing maternal protein restriction, especially the potential sex disparity in metabolic phenotype. Furthermore, the lack of activated autophagy-related gene expression in male offspring suggests that male offspring potentially have a reduced ability to adapt to a poor maternal environment^(47,48,49)^. As a result, males might have a greater risk of adverse outcomes^(49,50,51)^ and need more clinical attention than females. Understanding the sex disparity in physiological effects in offspring may help diagnose potential sex-biased diseases that occur in response to a maternal LP diet during pregnancy.

## References

[1] Jahan-Mihan A , Rodriguez J , Christie C , et al. (2015) The role of maternal dietary proteins in development of metabolic syndrome in offspring. Nutrients 7, 9185–9217. 26561832 10.3390/nu7115460 PMC4663588

[2] Wu G , Bazer FW , Cudd TA , et al. (2004) Maternal nutrition and fetal development. J Nutr 134, 2169–2172. 15333699 10.1093/jn/134.9.2169

[3] Fernandez-Twinn DS , Ozanne SE , Ekizoglou S , et al. (2003) The maternal endocrine environment in the low-protein model of intra-uterine growth restriction. Br J Nutr 90, 815–822. 13129451 10.1079/bjn2003967

[4] Morrison JL & Regnault TR (2016) Nutrition in pregnancy: optimising maternal diet and fetal adaptations to altered nutrient supply. Nutrients 8, 342. 10.3390/nu8060342 PMC4924183 27271666

[5] Zohdi V , Lim K , Pearson JT , et al. (2014) Developmental programming of cardiovascular disease following intrauterine growth restriction: findings utilising a rat model of maternal protein restriction. Nutrients 7, 119–152. 25551250 10.3390/nu7010119 PMC4303830

[6] Lillycrop KA , Phillips ES , Jackson AA , et al. (2005) Dietary protein restriction of pregnant rats induces and folic acid supplementation prevents epigenetic modification of hepatic gene expression in the offspring. J Nutr 135, 1382–1386. 15930441 10.1093/jn/135.6.1382

[7] Burdge GC , Slater-Jefferies J , Torrens C , et al. (2007) Dietary protein restriction of pregnant rats in the F0 generation induces altered methylation of hepatic gene promoters in the adult male offspring in the F1 and F2 generations. Br J Nutr 97, 435–439. 17313703 10.1017/S0007114507352392 PMC2211514

[8] Torres N , Bautista CJ , Tovar AR , et al. (2010) Protein restriction during pregnancy affects maternal liver lipid metabolism and fetal brain lipid composition in the rat. Am J Physiol Endocrinol Metab 298, E270–E277. 19920218 10.1152/ajpendo.00437.2009 PMC2822484

[9] Zheng J , Xiao X , Zhang Q , et al. (2017) Maternal low-protein diet modulates glucose metabolism and hepatic microRNAs expression in the early life of offspring dagger. Nutrients 9, 205. 10.3390/nu9030205 PMC5372868 28264458

[10] Harding HP , Novoa I , Zhang Y , et al. (2000) Regulated translation initiation controls stress-induced gene expression in mammalian cells. Mol Cell 6, 1099–1108. 11106749 10.1016/s1097-2765(00)00108-8

[11] B’Chir W , Maurin AC , Carraro V , et al. (2013) The eIF2α/ATF4 pathway is essential for stress-induced autophagy gene expression. Nucleic Acids Res 41, 7683–7699. 23804767 10.1093/nar/gkt563 PMC3763548

[12] Kilberg MS , Shan J & Su N (2009) ATF4-dependent transcription mediates signaling of amino acid limitation. Trends Endocrinol Metab 20, 436–443. 19800252 10.1016/j.tem.2009.05.008 PMC3587693

[13] Bruhat A , Jousse C , Wang XZ , et al. (1997) Amino acid limitation induces expression of CHOP, a CCAAT/enhancer binding protein-related gene, at both transcriptional and post-transcriptional levels. J Biol Chem 272, 17588–17593. 9211906 10.1074/jbc.272.28.17588

[14] Zhou D & Pan YX (2011) Gestational low protein diet selectively induces the amino acid response pathway target genes in the liver of offspring rats through transcription factor binding and histone modifications. Biochim Biophys Acta 1809, 549–556. 21777709 10.1016/j.bbagrm.2011.07.003

[15] Strakovsky RS , Zhou D & Pan YX (2010) A low-protein diet during gestation in rats activates the placental mammalian amino acid response pathway and programs the growth capacity of offspring. J Nutr 140, 2116–2120. 20980649 10.3945/jn.110.127803

[16] Wang H , Wilson GJ , Zhou D , et al. (2015) Induction of autophagy through the activating transcription factor 4 (ATF4)-dependent amino acid response pathway in maternal skeletal muscle may function as the molecular memory in response to gestational protein restriction to alert offspring to maternal nutrition. Br J Nutr 114, 519–532. 26198178 10.1017/S0007114515002172

[17] Mizushima N & Komatsu M (2011) Autophagy: renovation of cells and tissues. Cell 147, 728–741. 22078875 10.1016/j.cell.2011.10.026

[18] Maiuri MC , Zalckvar E , Kimchi A , et al. (2007) Self-eating and self-killing: crosstalk between autophagy and apoptosis. Nat Rev Mol Cell Biol 8, 741–752. 17717517 10.1038/nrm2239

[19] Zheng S , Rollet M & Pan YX (2012) Protein restriction during gestation alters histone modifications at the glucose transporter 4 (GLUT4) promoter region and induces GLUT4 expression in skeletal muscle of female rat offspring. J Nutr Biochem 23, 1064–1071. 22079207 10.1016/j.jnutbio.2011.05.013

[20] Chen H , Pan YX , Dudenhausen EE , et al. (2004) Amino acid deprivation induces the transcription rate of the human asparagine synthetase gene through a timed program of expression and promoter binding of nutrient-responsive basic region/leucine zipper transcription factors as well as localized histone acetylation. J Biol Chem 279, 50829–50839. 15385533 10.1074/jbc.M409173200

[21] Strakovsky RS , Wang H , Engeseth NJ , et al. (2015) Developmental bisphenol A (BPA) exposure leads to sex-specific modification of hepatic gene expression and epigenome at birth that may exacerbate high-fat diet-induced hepatic steatosis. Toxicol Appl Pharmacol 284, 101–112. 25748669 10.1016/j.taap.2015.02.021 PMC4520316

[22] Rouschop KM , van den Beucken T , Dubois L , et al. (2010) The unfolded protein response protects human tumor cells during hypoxia through regulation of the autophagy genes MAP1LC3B and ATG5. J Clin Invest 120, 127–141. 20038797 10.1172/JCI40027 PMC2798689

[23] Pike LR , Singleton DC , Buffa F , et al. (2013) Transcriptional up-regulation of ULK1 by ATF4 contributes to cancer cell survival. Biochem J 449, 389–400. 23078367 10.1042/BJ20120972

[24] Raciti M , Lotti LV , Valia S , et al. (2012) JNK2 is activated during ER stress and promotes cell survival. Cell Death Dis 3, e429. 23171849 10.1038/cddis.2012.167 PMC3542603

[25] Huang J & Klionsky DJ (2007) Autophagy and human disease. Cell Cycle 6, 1837–1849. 17671424 10.4161/cc.6.15.4511

[26] Kraft C & Martens S (2012) Mechanisms and regulation of autophagosome formation. Curr Opin Cell Biol 24, 496–501. 22664348 10.1016/j.ceb.2012.05.001

[27] Kang R , Zeh HJ , Lotze MT , et al. (2011) The Beclin 1 network regulates autophagy and apoptosis. Cell Death Differ 18, 571–580. 21311563 10.1038/cdd.2010.191 PMC3131912

[28] Nakatogawa H (2013) Two ubiquitin-like conjugation systems that mediate membrane formation during autophagy. Essays Biochem 55, 39–50. 24070470 10.1042/bse0550039

[29] Shpilka T , Mizushima N & Elazar Z (2012) Ubiquitin-like proteins and autophagy at a glance. J Cell Sci 125, 2343–2348. 22736434 10.1242/jcs.093757

[30] Pankiv S , Clausen TH , Lamark T , et al. (2007) p62/SQSTM1 binds directly to Atg8/LC3 to facilitate degradation of ubiquitinated protein aggregates by autophagy. J Biol Chem 282, 24131–24145. 17580304 10.1074/jbc.M702824200

[31] Dearden L , Bouret SG & Ozanne SE (2018) Sex and gender differences in developmental programming of metabolism. Mol Metab 15, 8–19. 29773464 10.1016/j.molmet.2018.04.007 PMC6066743

[32] Lista P , Straface E , Brunelleschi S , et al. (2011) On the role of autophagy in human diseases: a gender perspective. J Cell Mol Med 15, 1443–1457. 21362130 10.1111/j.1582-4934.2011.01293.x PMC3823190

[33] Vitale C , Mendelsohn ME & Rosano GM (2009) Gender differences in the cardiovascular effect of sex hormones. Nat Rev Cardiol 6, 532–542. 19564884 10.1038/nrcardio.2009.105

[34] Vega-Naredo I , Caballero B , Sierra V , et al. (2009) Sexual dimorphism of autophagy in Syrian hamster Harderian gland culminates in a holocrine secretion in female glands. Autophagy 5, 1004–1017. 19736526 10.4161/auto.5.7.9610

[35] Campesi I , Straface E , Occhioni S , et al. (2013) Protein oxidation seems to be linked to constitutive autophagy: a sex study. Life Sci 93, 145–152. 23770210 10.1016/j.lfs.2013.06.001

[36] Jia Y , Cong R , Li R , et al. (2012) Maternal low-protein diet induces gender-dependent changes in epigenetic regulation of the glucose-6-phosphatase gene in newborn piglet liver. J Nutr 142, 1659–1665. 22833655 10.3945/jn.112.160341

[37] Kwong WY , Miller DJ , Wilkins AP , et al. (2007) Maternal low protein diet restricted to the preimplantation period induces a gender-specific change on hepatic gene expression in rat fetuses. Mol Reprod Dev 74, 48–56. 16941667 10.1002/mrd.20606

[38] Mohapatra S , Chakraborty T , Shimizu S , et al. (2020) Estrogen and estrogen receptors chauffeur the sex-biased autophagic action in liver. Cell Death Differ 27, 3117–3130. 32483382 10.1038/s41418-020-0567-3 PMC7566502

[39] Xiang J , Liu X , Ren J , et al. (2019) How does estrogen work on autophagy? Autophagy 15, 197–211. 30208759 10.1080/15548627.2018.1520549 PMC6333457

[40] Toledo FC , Perobelli JE , Pedrosa FP , et al. (2011) In utero protein restriction causes growth delay and alters sperm parameters in adult male rats. Reprod Biol Endocrinol 9, 94. 21702915 10.1186/1477-7827-9-94 PMC3141647

[41] Zambrano E , Rodriguez-Gonzalez GL , Guzman C , et al. (2005) A maternal low protein diet during pregnancy and lactation in the rat impairs male reproductive development. J Physiol 563, 275–284. 15611025 10.1113/jphysiol.2004.078543 PMC1665555

[42] Shen M & Shi H (2015) Sex hormones and their receptors regulate liver energy homeostasis. Int J Endocrinol 2015, 294278. 26491440 10.1155/2015/294278 PMC4600502

[43] Maselli A , Matarrese P , Straface E , et al. (2009) Cell sex: a new look at cell fate studies. FASEB J 23, 978–984. 19074513 10.1096/fj.08-114348

[44] Qu X , Yu J , Bhagat G , et al. (2003) Promotion of tumorigenesis by heterozygous disruption of the Beclin 1 autophagy gene. J Clin Invest 112, 1809–1820. 14638851 10.1172/JCI20039 PMC297002

[45] Singh R , Kaushik S , Wang Y , et al. (2009) Autophagy regulates lipid metabolism. Nature 458, 1131–1135. 19339967 10.1038/nature07976 PMC2676208

[46] Koenig A , Sateriale A , Budd RC , et al. (2014) The role of sex differences in autophagy in the heart during Coxsackie virus B_3_-induced myocarditis. J Cardiovasc Transl Res 7, 182–191. 24323874 10.1007/s12265-013-9525-5 PMC4115281

[47] Cheong JN , Wlodek ME , Moritz KM , et al. (2016) Programming of maternal and offspring disease: impact of growth restriction, fetal sex and transmission across generations. J Physiol 594, 4727–4740. 26970222 10.1113/JP271745 PMC5009791

[48] Bromfield JJ , Schjenken JE , Chin PY , et al. (2014) Maternal tract factors contribute to paternal seminal fluid impact on metabolic phenotype in offspring. Proc Natl Acad Sci U S A 111, 2200–2205. 24469827 10.1073/pnas.1305609111 PMC3926084

[49] Isganaitis E , Woo M , Ma H , et al. (2014) Developmental programming by maternal insulin resistance: hyperinsulinemia, glucose intolerance, and dysregulated lipid metabolism in male offspring of insulin-resistant mice. Diabetes 63, 688–700. 24186867 10.2337/db13-0558 PMC3900545

[50] Gawlinska K , Gawlinski D , Korostynski M , et al. (2021) Maternal dietary patterns are associated with susceptibility to a depressive-like phenotype in rat offspring. Dev Cogn Neurosci 47, 100879. 33232913 10.1016/j.dcn.2020.100879 PMC7691544

[51] Spearman AD , Ke X , Fu Q , et al. (2018) Adverse maternal environment leads to cardiac fibrosis in adult male mice. Birth Defects Res 110, 1551–1555. 30576090 10.1002/bdr2.1428

